# Characteristics and research waste of immunotherapy randomized clinical trials: a cross-sectional analysis

**DOI:** 10.3389/fimmu.2026.1845591

**Published:** 2026-08-17

**Authors:** Jiyang Li, Shiqi Wang, Qiuyu Zhou, Weiyi Li, Zhangyu Xu, Xinxing Fei, Yue Hu

**Affiliations:** 1 Department of Rehabilitation Medicine, Sichuan Tianfu New Area People’s Hospital, Chengdu, China; 2 Rehabilitation Medicine Center and Institute of Rehabilitation Medicine, West China Hospital, Sichuan University, Chengdu, Sichuan, China; 3 Key Laboratory of Rehabilitation Medicine in Sichuan Province, Chengdu, Sichuan, China; 4 Department of Rehabilitation Medicine, The Affiliated Hospital of Southwest Medical University, Luzhou, China; 5 Rehabilitation Medicine and Engineering Key Laboratory of Luzhou, Luzhou, China; 6 Department of Psychiatry, Chengdu Eighth People’s Hospital (Geriatric Hospital of Chengdu Medical College), Chengdu, China

**Keywords:** clinical trial methodology, evidence-based medicine, immunotherapy, randomized controlled trial, research waste

## Abstract

**Background:**

Immunotherapy spans tumor and non-tumor disciplines, yet the surge in randomized controlled trials (RCTs) has not yielded proportional improvements in evidence quality, leading to substantial research waste. In this study, the immunotherapy RCT landscape was characterized, and the prevalence and risk factors for research waste were quantified.

**Methods:**

This registry-based cross-sectional study analyzed phase III/IV immunotherapy RCTs from ClinicalTrials.gov. The publication status was verified via PubMed, Scopus, and Google Scholar. Research waste was defined as non-publication, inadequate reporting (CONSORT 2010), or avoidable design flaws (Cochrane Risk of Bias Tool). Associations between study characteristics and outcomes (publication status and waste) were evaluated using logistic regression.

**Results:**

Among the 164 RCTs analyzed, 127 (77.4%) exhibited research waste. With respect to publication status, 74 (45.1%) were published, and 90 (54.9%) were unpublished. Multivariate analysis revealed that non-tumor diseases (adjusted OR = 0.29, 95% CI: 0.09–0.85, P = 0.028) and a quadruple-blind design (adjusted OR = 4.84, 95% CI: 1.71–14.90, P = 0.004) were significantly associated with publication status. Univariable analysis revealed that non-tumor diseases (OR = 4.36, 95% CI: 2.02–9.57, P<0.001) and European principal investigators (PIs) (OR = 3.48, 95% CI: 1.49–8.16, P = 0.004) were positively correlated with waste, whereas international recruitment (OR = 0.16, 95% CI: 0.07–0.36, P<0.001), multicenter design (OR = 0.14, 95% CI: 0.05–0.35, P<0.001), and large sample size (OR = 0.27, 95% CI: 0.12–0.60, P = 0.001) were negatively correlated. After adjustment, non-tumor diseases remained a risk factor (OR = 3.84, 95% CI: 1.18–13.54, P = 0.029), whereas a multicenter design had a strong protective effect (OR = 0.07, 95% CI: 0.00–0.57, P = 0.030).

**Conclusions:**

This study revealed a high incidence of research waste in immunotherapy RCTs, which was predominantly driven by non-tumor studies. These findings underscore the urgent need to prioritize large-scale collaborative frameworks to optimize resource allocation and ensure reliable clinical evidence.

## Introduction

Immunotherapy comprises therapeutic interventions designed to modulate the host immune response to achieve clinical benefit (1, 2). While this approach is firmly established in oncology, where immune checkpoint inhibitors have become a cornerstone of modern cancer care, it is also extensively utilized in non-neoplastic disciplines (3). Immunomodulatory strategies are routinely employed in the management of conditions such as allergic rhinitis, asthma, and various autoimmune disorders across otorhinolaryngological, respiratory, and dermatological specialties (4–6). This widespread application across both tumor and non-tumor diseases has led to a substantial increase in randomized controlled trials (RCTs) evaluating immunotherapeutic interventions, necessitating a critical evaluation of their methodological quality and translational efficiency (7).

However, the surge in RCTs has not necessarily led to a concomitant improvement in the quality of evidence, and research waste has become a hidden barrier to the efficient development of evidence-based medicine (8). Research waste typically manifests as unpublished trial data, inadequate reporting, or avoidable design flaws, which not only lead to the ineffective use of vast research funds and participant resources but also impede the timely translation of effective medical interventions into clinical practice (9, 10). Given that immunotherapy RCTs often involve complex biomarker enrichment designs, long-term survival follow-up, and expensive biological costs, the potential risk of waste and its consequences in this field may be far more severe than those in conventional drug trials (11, 12); however, there is currently a lack of systematic quantitative analysis based on a broad disease spectrum.

Therefore, this study aimed to comprehensively characterize the spatiotemporal distribution and design features of registered immunotherapy RCTs since the early 2000s through a cross-sectional analysis, focusing on evaluating waste in terms of publication status, reporting quality, and design rigor. Unlike previous studies focusing on individual diseases, this project examined the differential performance of immunotherapy across tumor and non-tumor specialties, aiming to provide more generalizable, evidence-based guidance to optimize global clinical trial return on investment and resource allocation.

## Methods

### Study design and data source

This registry-based cross-sectional study was designed to characterize immunotherapy RCTs and assess research waste. Data were sourced from the internationally recognized clinical trial registry, ClinicalTrials.gov. Given that this study involved only a secondary analysis of publicly available registration information and did not involve patient privacy or biological sample collection, ethical approval and informed consent were not needed. Reporting followed the Strengthening the Reporting of Observational Studies in Epidemiology (STROBE) guideline.

### Search strategy and study selection

We performed a systematic search in ClinicalTrials.gov. The search was restricted to interventional studies with the keyword “immunotherapy,” which were limited to completed phase III and IV trials involving adult participants. We focused on phase III and IV trials because they provide pivotal, post-marketing evidence with the highest methodological maturity and greatest potential for clinical impact; early-phase (I/II) trials were excluded to avoid confounding by higher discontinuation rates and exploratory endpoints. To mitigate time lag bias in assessing publication status, we applied a dual cutoff: trials were eligible if they started between January 1, 2000, and December 31, 2018, or completed on or before December 31, 2023. These cutoffs were informed by empirical data indicating a median interval of approximately 5 years from trial initiation to completion and an additional 2 years from completion to full-text publication, thereby providing sufficient opportunity for dissemination while maintaining contemporary relevance (13).

### Variable definition and data extraction

Two independent investigators imported the search results into a standardized Excel database and extracted the data; discrepancies were resolved through discussion with a third investigator. The extracted variables included the following: (1) Basic characteristics: registration year; disease type (tumor and non-tumor); primary purpose (treatment, prevention, and other); study design (parallel and non-parallel groups); number of arms; blinding method; recruitment scope; number of centers; number of participants (divided into large and small sample sizes based on the upper quartile of participant numbers); region of principal investigator (PI); and funding source (industry and non-industry). Blinding status was extracted from ClinicalTrials.gov. Based on registry descriptors and aligned with the Cochrane Risk of Bias 2.0 framework, “quadruple-blind” was defined as the masking of participants, care providers, investigators, and outcome assessors to treatment allocation (14). (2) Publication status: The PubMed, Scopus, and Google Scholar databases were searched using the NCT number and PI name to confirm whether the trial had been published as a full text in a peer-reviewed journal. Trials marked as “Completed” in ClinicalTrials.gov but for which no corresponding full-text publication was found before the cutoff date were considered unpublished. (3) Adequate reporting status: Published RCTs were evaluated using key items of the CONSORT 2010 checklist. Consistent with prior studies, a threshold of ≥75% completeness was adopted to define adequate reporting, balancing sensitivity and specificity for identifying meaningful reporting gaps while accounting for variability in item applicability across trials (15). Although CONSORT 2025 has been released, considering that all the included RCTs were published before 2025, we continued to use the 2010 version for the assessment. (4) Avoidable design flaws: The Cochrane Risk of Bias Tool was used to evaluate the trial design. A third investigator reconciled independent assessments by two investigators to reach consensus.​ Any item assessed as “High risk” or “Unclear risk” was defined as having a design defect, covering dimensions such as random sequence generation, allocation concealment, performance bias, detection bias, attrition bias, and reporting bias. (5) Research waste assessment: Research waste was defined in accordance with established frameworks as the failure to maximize the utility of research investments. In this study, waste was operationalized as the presence of at least one of the following: non-publication, inadequate reporting, or avoidable design flaws (16, 17).

### Statistical analysis

Descriptive statistics were presented for the characteristics of immunotherapy RCTs. Chi-square tests or Fisher’s exact tests were used to compare categorical variables. Logistic regression was performed to explore the associations between study characteristics and outcome indicators (publication status and waste status). In univariable logistic regression, missing values were handled using pairwise deletion: each covariate was analyzed using all RCTs with available data for that specific factor, resulting in varying sample sizes across predictors. Multivariable models were constructed using *a priori*–defined covariates encompassing all extractable experimental design features (18).​ Given the exploratory nature of this study, all the variables were subsequently entered into the multivariable logistic regression model regardless of their univariable significance. A complete-case analysis was applied in the multivariable model, which included only RCTs with complete data across all covariates. Variables were entered using the Enter method. All the statistical analyses were conducted using Prism (version 10.1.2), and a two-sided P value <0.05 was considered statistically significant. Forest plots were generated using the Mengteyun statistical platform.

## Results

### Basic characteristics of included RCTs

**Figure 1:**
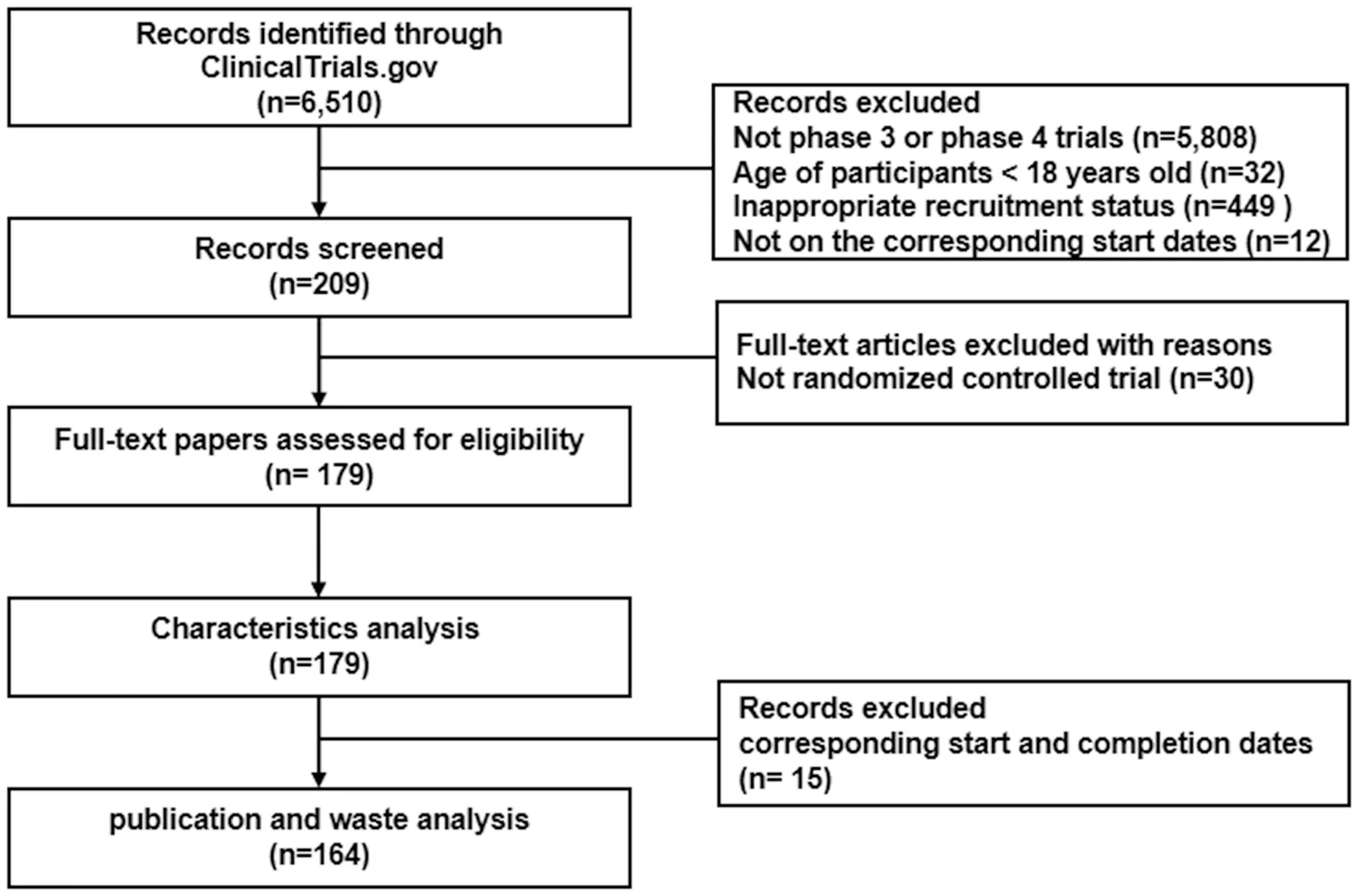
Flow diagram.

**Figure 2:**
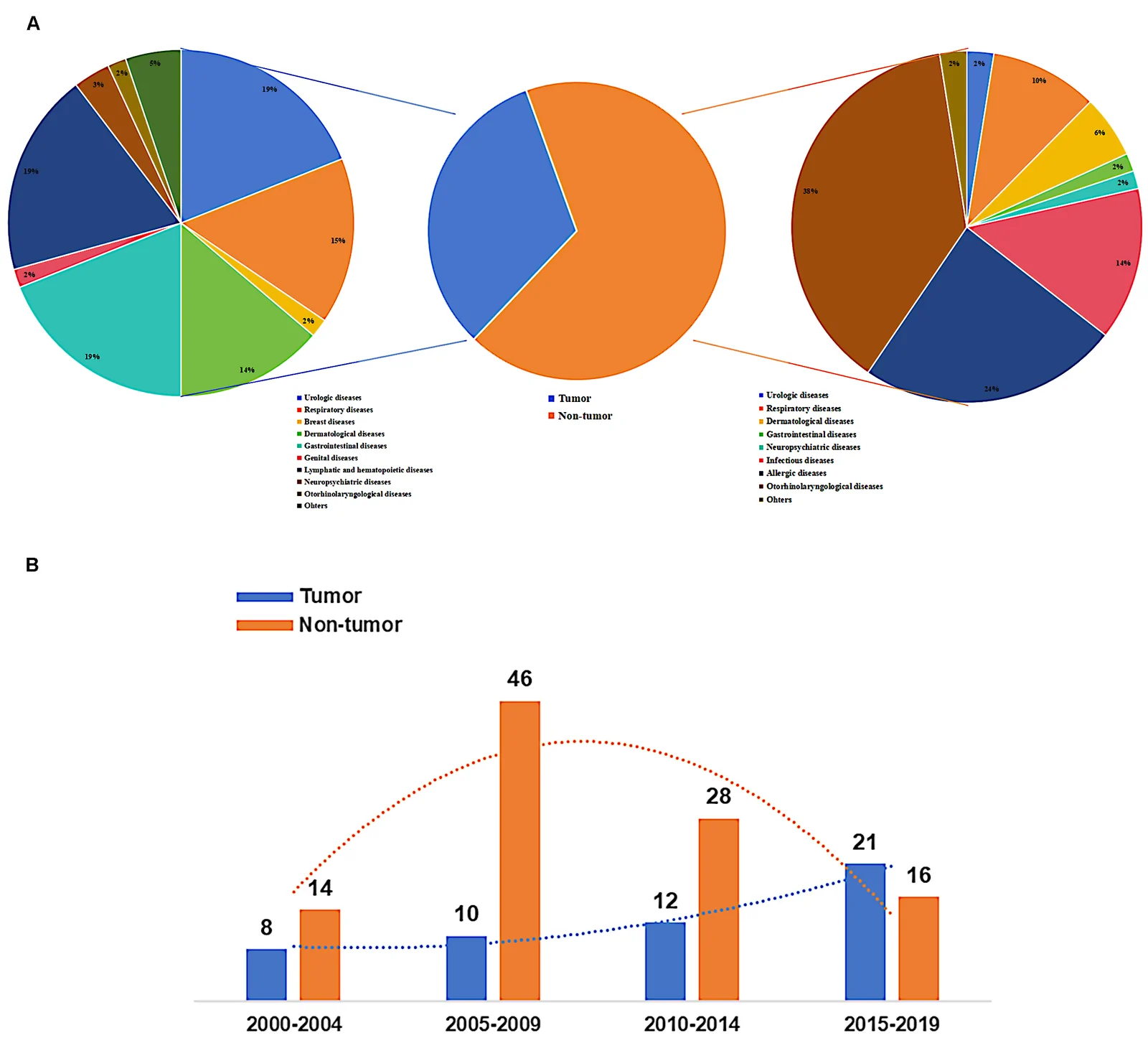
Distribution and temporal trends of immunotherapy RCTs by disease type. **(A)**​ Pie charts showing the overall distribution of eligible RCTs (left), the proportion of tumor and non-tumor studies (middle), and the breakdown of non-tumor RCTs by disease category (right). **(B)**​ Annual trends of tumor (blue) and non-tumor (orange) RCTs from 2000 to 2019. Non-tumor trials peaked in 2005–2009, whereas tumor trials showed a gradual increase over time.

A total of 179 RCTs were included in the characteristic analysis (**Figure 1**). The temporal distribution revealed that registrations peaked during 2005–2009 (56 trials, 31.3%) and then fluctuated downward (**Table 1**). In terms of disease type, non-tumor-related studies accounted for a significantly higher proportion than tumor-related studies did, with most focusing on otorhinolaryngological diseases, particularly allergic rhinitis. In contrast, the proportion of tumor immunotherapy studies increased over time, with a focus on the urologic, gastrointestinal, lymphatic, and hematopoietic systems (**Figure 2**). In terms of study design, the vast majority adopted a parallel group design (172 trials, 96.1%) and were treatment oriented (163 trials, 91.1%), primarily consisting of two-arm studies (147 trials, 82.1%). Notably, although open-label trials accounted for a certain proportion (54 trials, 30.2%), double-blind (48 trials, 26.8%) and quadruple-blind (49 trials, 27.4%) designs were also widely applied. Furthermore, multicenter studies (103 trials, 57.5%) and trials with a participant scale <550 (132 trials, 73.7%) were dominant, with PIs mostly from Europe (113 trials, 63.1%) and funding sources mainly from industry (108 trials, 60.3%). Among these RCTs, the median planned sample size was 229 (interquartile range: 96–549). Thus, a threshold of 550 participants—corresponding to the upper quartile—was used to categorize trials as having small or large sample sizes.

**Table 1:** Characteristics of registered RCTs related to immunotherapy.

Characteristics	n = 179	No. (%)
Start date		
2000-2004	22	12.3%
2005-2009	56	31.3%
2010-2014	40	22.4%
2015-2019	37	20.7%
After 2019	24	13.4%
Conditions		
Tumor	58	32.4%
Non-tumor	121	67.6%
Primary purpose		
Treatment	163	91.1%
Prevention	11	6.2%
Other	5	2.8%
Study design		
Parallel group	172	96.1%
Non-Parallel group	7	3.9%
No. of arms		
2	147	82.1%
3	17	9.5%
≥4	15	8.4%
Blinding		
None/open label	54	30.2%
Single	6	3.4%
Double	48	26.8%
Triple	22	12.3%
Quadruple	49	27.4%
Recruitment		
National	111	62.0%
International	66	36.9%
Missing	2	1.1%
No. of centers		
Single center	74	41.3%
Multicenter	103	57.5%
Missing	2	1.1%
No. of participants		
<550	132	73.7%
≥550	43	24.0%
Missing	11	6.2%
Region of PI		
North America	46	25.7%
Europe	113	63.1%
Asia	12	6.7%
Others	8	4.5%
Funding		
Industry	108	60.3%
Non-industry	71	39.7%

PI, principal investigator.

### Publication status

Among the 164 RCTs included in the publication and waste analysis, 74 (45.1%) were published, and 90 (54.9%) were unpublished. Significant differences were observed in the distribution of disease type (P = 0.003), recruitment scope (P<0.001), and participant number (P = 0.001) across different publication statuses. Specifically, the proportions of tumor diseases, international recruitment, and large sample sizes were significantly higher in the published group than in the unpublished group. No statistically significant differences were found in terms of the registration year, study design, blinding, number of centers, PI region, or funding source (P>0.05) (**Table 2**).

**Table 2:** Characteristics of RCTs related to immunotherapy with different publication status.

Characteristic	Published (n=74)	No. (%)	Not published (n=90)	No. (%)	P
Start date					0.592
2000-2004	9	12.2%	13	14.4%	
2005-2009	22	29.7%	34	37.8%	
2010-2014	22	29.7%	18	20.0%	
2015-2019	15	20.3%	16	17.8%	
After 2019	6	8.1%	9	10.0%	
Conditions					0.003
Tumor	30	40.5%	17	18.9%	
Non-tumor	44	59.5%	73	81.1%	
Primary purpose					0.587
Treatment	68	91.9%	81	90.0%	
Prevention	5	6.8%	5	5.6%	
Other	1	1.4%	4	4.4%	
Study design					0.659
Parallel group	71	96.0%	88	97.8%	
Non-Parallel group	3	4.1%	2	2.2%	
No. of arms					0.633
2	59	79.7%	76	84.4%	
3	9	12.2%	7	7.8%	
≥4	6	8.1%	7	7.8%	
Blinding					0.155
None/open label	18	24.3%	30	33.3%	
Single	3	4.1%	2	2.2%	
Double	17	23.0%	28	31.1%	
Triple	8	10.8%	11	12.2%	
Quadruple	28	37.8%	19	21.1%	
Recruitment					<0.001
National	35	47.3%	66	73.3%	
International	39	52.7%	22	24.4%	
Missing	0	0.0%	2	2.2%	
No. of centers					0.050
Single center	24	32.4%	37	41.1%	
Multicenter	50	67.6%	41	45.6%	
Missing	0	0.0%	2	2.2%	
No. of participants					0.001
<550	47	63.5%	73	81.1%	
≥550	27	36.5%	13	14.4%	
Missing	0	0.0%	4	4.4%	
Region of PI					0.559
North America	20	27.0%	16	17.8%	
Europe	46	62.2%	64	71.1%	
Asia	5	6.8%	6	6.7%	
Others	3	4.1%	4	4.4%	
Funding					0.523
Industry	43	58.1%	57	63.3%	
Non-industry	31	41.9%	33	36.7%	

PI, principal investigator.

**Figure 3:**
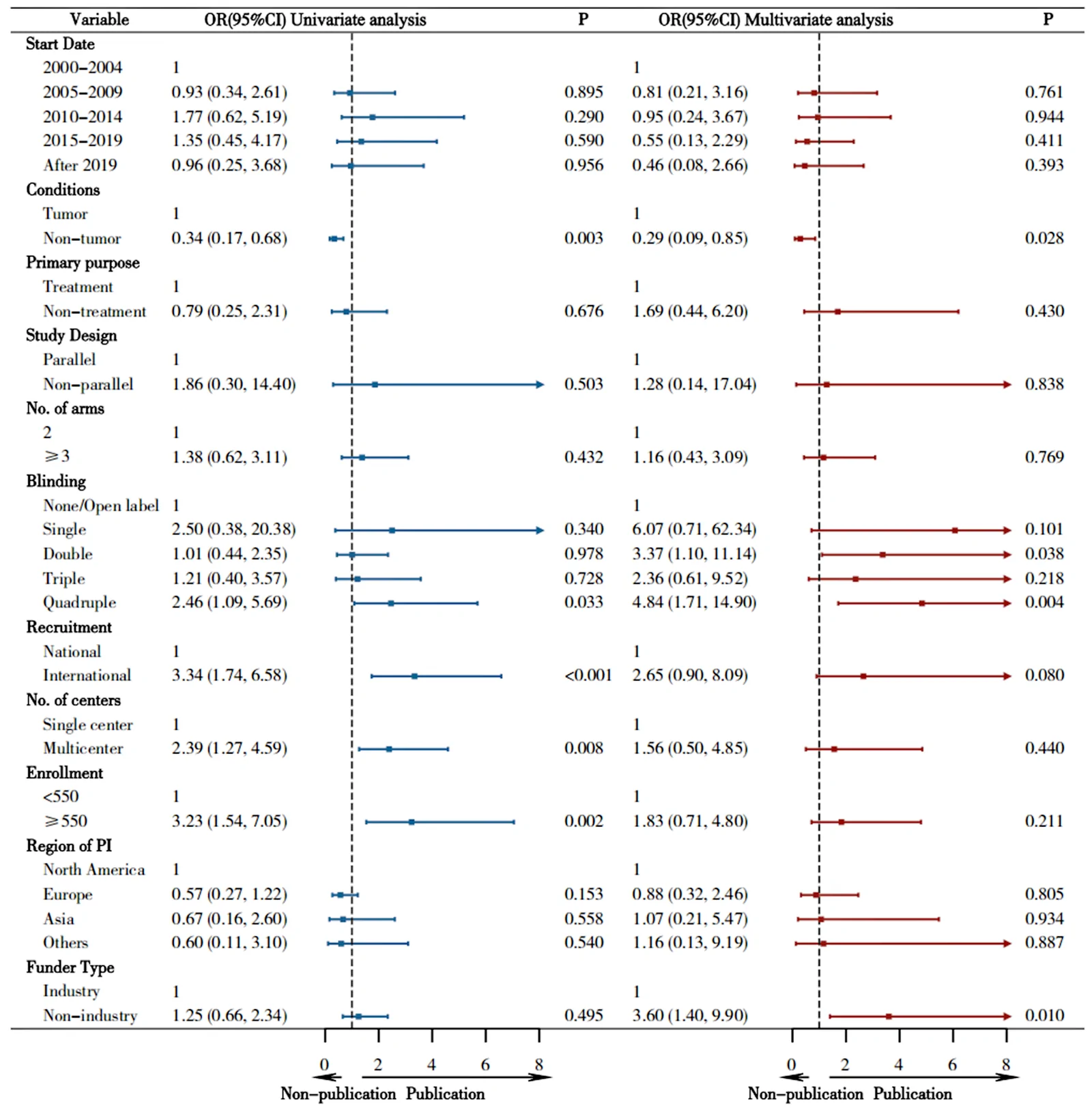
Logistic regression analysis of factors associated with publication status. Forest plots display univariate (left) and multivariate (right) ORs with 95% CIs for factors associated with publication. Multivariate models adjusted for all listed covariates. Sample sizes varied in univariate analyses because of pairwise deletion (164 for most, 162 for Recruitment/centers, and 160 for Enrollment). The multivariate analysis included 160 RCTs with complete data across all covariates.

We further constructed logistic regression models to quantify the strength of the association between study characteristics and publication status. Univariate analysis revealed that non-tumor diseases (OR = 0.34, 95% CI: 0.17–0.70, P = 0.003) and a quadruple-blind design (OR = 2.46, 95% CI: 1.09–5.69, P = 0.033) were significantly associated with a lower likelihood of publication. International recruitment (OR = 3.34, 95% CI: 1.74–6.58, P<0.001), multicenter studies (OR = 2.39, 95% CI: 1.27–4.59, P = 0.008), and a sample size ≥550 (OR = 3.23, 95% CI: 1.54–7.05, P = 0.0002) were significantly associated with increased publication likelihood. However, in the multivariable model, only non-tumor diseases (adjusted OR = 0.29, 95% CI: 0.09–0.85, P = 0.028), a quadruple-blind design (adjusted OR = 4.84, 95% CI: 1.71–14.90, P = 0.004), and non-industry funding (adjusted OR = 3.60, 95% CI: 1.40–9.90, P = 0.010) were significantly independently associated with publication status (**Figure 3**).

### Reporting completeness

Among the 74 published articles, 62 (83.8%) met the adequate reporting standard, while 12 (16.2%) were inadequately reported. Only the number of participants significantly differed between groups (P = 0.046), with a higher proportion of inadequately reported trials in the < 550-sample-size group. No other characteristics were significantly different (P>0.05) (**Supplementary Table 1**). Additionally, most RCTs reported core CONSORT items well. However, significant omissions remained regarding specific methodological details, particularly Item 24 (trial protocol access), reported by only 31.1% of the literature; reporting rates for Item 8a (method of random sequence generation) and Item 9 (allocation implementation mechanism) were also low, at 64.9% and 63.5%, respectively (**Supplementary Table 2**).

### Design flaw

**Figure 4:**
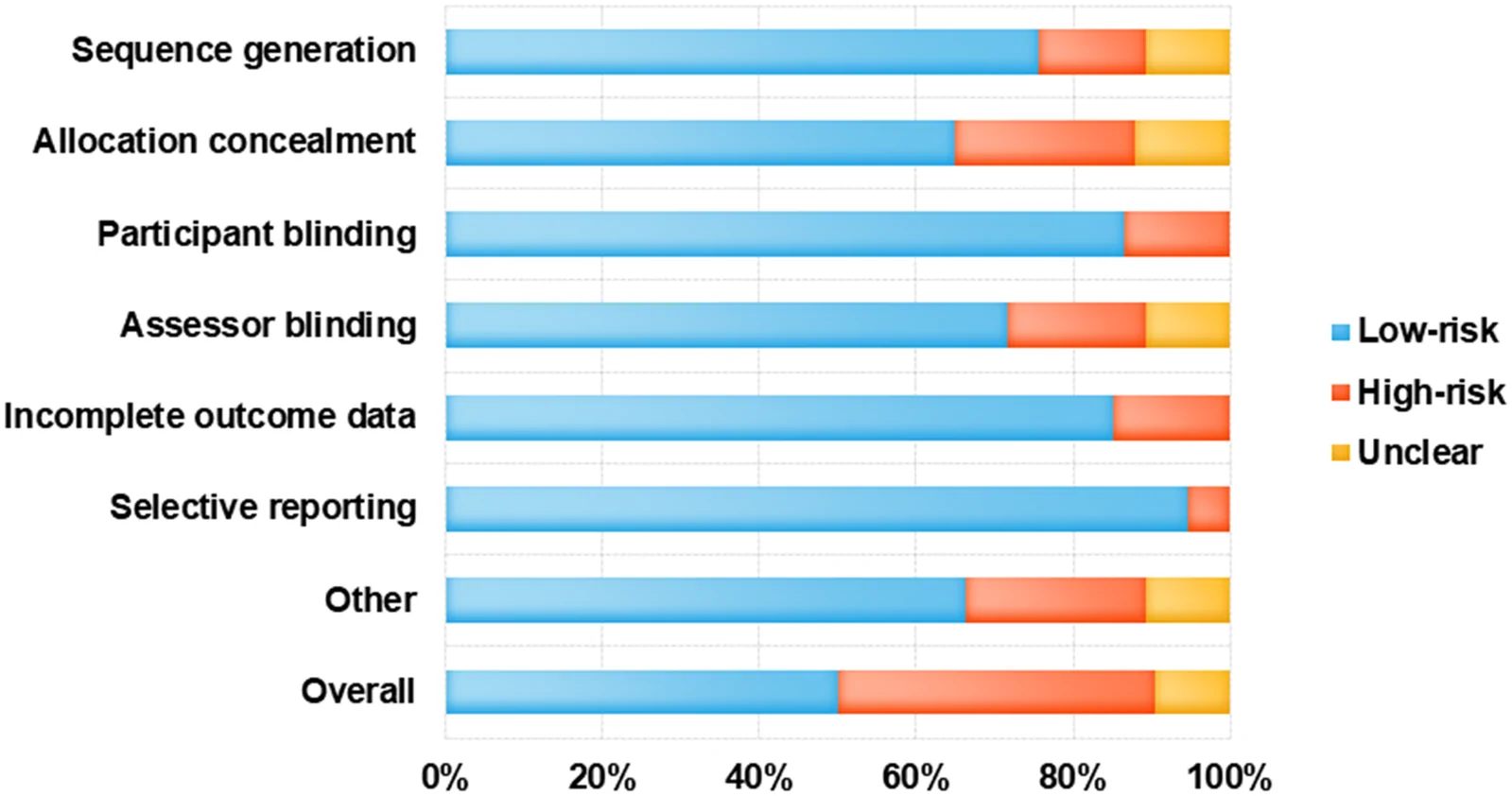
Risk of bias assessment across published RCTs.

Among the 74 published RCTs, exactly half (37 trials, 50.0%) had at least one avoidable design flaw (**Supplementary Table 3**). The number of trials with design flaws was more concentrated in non-tumor diseases (P = 0.032), domestic recruitment (P = 0.005), and single-center studies (P = 0.001). Moreover, trials with PIs from Europe also showed a significantly higher proportion of design flaws (P = 0.016) (**Supplementary Table 3**). Furthermore, although low risk accounted for a high proportion of “incomplete outcome data,” “selective reporting,” and “blinding implementation,” the high-risk proportion remained prominent in “allocation concealment” and “random sequence generation,” with an overall high-risk rate of approximately 40.54% (**Figure 4**; **Supplementary Table 4**).

### Waste status

Among the 164 immunotherapy-related RCTs analyzed, 127 (77.4%) exhibited at least one form of research waste, and only 37 (22.6%) showed no research waste (**Supplementary Table 5**). Disease type (P<0.001), recruitment scope (P<0.001), number of centers (P<0.001), sample size (P = 0.003), and PI region (P = 0.010) significantly differed between the groups (**Table 3**). Specifically, the proportions of trials involving non-tumor diseases, domestic recruitment, single-center studies, a sample size <550, and PIs from Europe were significantly greater. The other characteristics did not significantly differ (P>0.05) (**Table 3**).

**Table 3:** Characteristics of RCTs related to immunotherapy regarding the situation of waste.

Characteristic	Without research waste (n=37)	No. (%)	With research waste (n=127)	No. (%)	P
Start date					0.072
2000-2004	1	2.7%	21	16.5%	
2005-2009	10	27.0%	46	36.2%	
2010-2014	12	32.4%	28	22.1%	
2015-2019	10	27.0%	21	16.5%	
After 2019	4	10.8%	11	8.7%	
Conditions					<0.001
Tumor	20	54.1%	27	21.3%	
Non-tumor	17	45.9%	100	78.7%	
Primary purpose					0.686
Treatment	35	94.6%	114	89.8%	
Prevention	2	5.4%	8	6.3%	
Other	0	0.0%	5	3.9%	
Study design					0.315
Parallel group	35	94.6%	124	97.6%	
Non-Parallel group	2	5.4%	3	2.4%	
No. of arms					0.874
2	31	83.8%	104	81.9%	
3	4	10.8%	12	9.5%	
≥4	2	5.4%	11	8.7%	
Blinding					0.108
None/open label	10	27.0%	38	29.9%	
Single	2	5.4%	3	2.4%	
Double	5	13.5%	40	31.5%	
Triple	6	16.2%	13	10.2%	
Quadruple	14	37.8%	33	26.0%	
Recruitment					<0.001
National	11	29.7%	90	70.9%	
International	26	70.3%	35	27.6%	
Missing	0	0.0%	2	1.6%	
No. of centers					<0.001
Single center	5	13.5%	66	52.0%	
Multicenter	32	86.5%	59	46.5%	
Missing	0	0.0%	2	1.6%	
No. of participants					0.003
<550	20	54.1%	100	78.7%	
≥550	17	45.9%	23	18.1%	
Missing	0	0.0%	4	3.2%	
Region of PI					0.010
North America	14	37.8%	22	17.3%	
Europe	17	45.9%	93	73.2%	
Asia	3	8.1%	8	6.3%	
Others	3	8.1%	4	3.2%	
Funding					0.251
Industry	26	70.3%	74	58.3%	
Non-industry	11	29.7%	53	41.7%	

PI, principal investigator.

**Figure 5:**
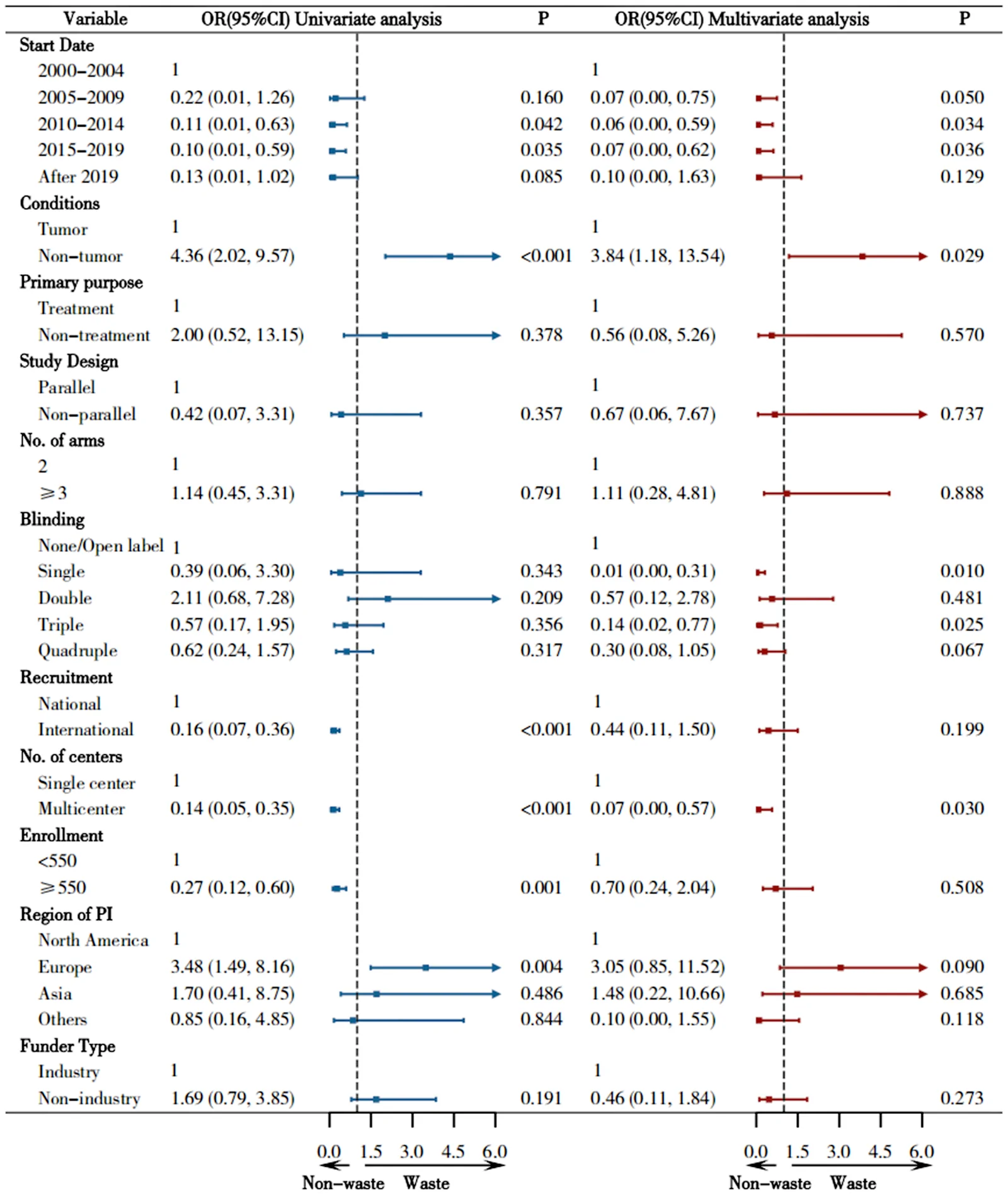
Logistic regression of factors associated with research waste. Forest plots display univariate (left) and multivariate (right) ORs with 95% CIs for factors associated with publication. Multivariate models adjusted for all listed covariates. Sample sizes varied in univariate analyses because of pairwise deletion (164 for most, 162 for Recruitment/centers, and 160 for Enrollment). The multivariate analysis included 160 RCTs with complete data across all covariates.

We further constructed logistic regression models to quantify the strength of the associations between study characteristics and research waste (**Figure 5**). Univariate analysis revealed that non-tumor disease studies (OR = 4.36, 95%CI: 2.02–9.57, P<0.001) and European PIs (OR = 3.48, 95%CI: 1.49–8.16, P = 0.004) were positively correlated with research waste; whereas international recruitment (OR = 0.16, 95%CI: 0.07–0.36, P<0.001), multicenter design (OR = 0.14, 95%CI: 0.05–0.35, P<0.001), and large sample size (≥550) (OR = 0.27, 95%CI: 0.12–0.60, P = 0.001) were negatively correlated with research waste.

After adjusting for potential confounders in the multivariable analysis, non-tumor disease studies still showed a significant positive correlation with research waste (OR = 3.84, 95%CI: 1.18–13.54, P = 0.029). Multicenter design (OR = 0.07, 95%CI: 0.00–0.57, P = 0.030), single-blind design (OR = 0.01, 95%CI: 0.00–0.31, P = 0.010), and triple-blind design (OR = 0.14, 95%CI: 0.02–0.77, P = 0.025) remained significantly negatively correlated with research waste. Furthermore, compared with the early period, studies starting between 2010–2014 (OR = 0.06, 95%CI: 0.00–0.59, P = 0.034) and 2015–2019 (OR = 0.07, 95%CI: 0.00–0.62, P = 0.036) showed stronger protective trends.

## Discussion

Our cross-sectional analysis of relevant registered RCTs revealed the structural distribution patterns of immunotherapy clinical trials within a specific timeframe. The data indicate that the proportion of trials in non-tumor fields is significantly higher than that in tumor fields, primarily because of the large number of immunotherapy clinical trials for allergic diseases (19, 20). Such trials usually feature well-defined disease courses, standardized symptom scoring scales, and mature placebo-controlled models, making them readily identifiable in registries. Furthermore, the dominance of non-industry funding suggests that a significant portion of research in this field is not investigator-initiated trials (IITs) but is conducted by pharmaceutical companies as confirmatory phase III or phase IV trials. This industry dominance underscores that commercial imperatives may largely drive the evaluation of immunotherapy to secure regulatory approval rather than purely by academic exploration.

The low publication rate of non-tumor disease trials cannot be attributed solely to negative results. Rather, these findings may reflect a predominance of confirmatory studies for established immunotherapies, such as allergen immunotherapy. Thus, incremental improvements in symptom scores—while clinically meaningful—may lack the transformative scientific narrative often required for high-impact dissemination. This potential disconnect between clinical utility and perceived academic novelty could create a publication barrier, irrespective of trial quality or outcomes (5, 21). However, ascertaining the precise reasons for non-publication lies beyond the scope of registry-based analyses, as we could not access internal sponsor decisions, peer-review feedback, or investigators’ intentions.​ While surveys, Delphi panels, or focus groups are ideal for elucidating these underlying drivers, such primary data collection was outside the purview of this study. Future research employing these qualitative methods is warranted to validate hypothesized barriers to dissemination. Conversely, immunotherapy trials in oncology, even when yielding negative results, are more frequently disseminated because of the substantial clinical implications of survival outcomes.

Notably, quadruple-blind trials were associated with a higher likelihood of publication. This finding likely reflects that, in the absence of access to full trial protocols during peer review, complex blinding procedures serve as a salient proxy for overall methodological rigor (22). Editors and reviewers may reasonably infer that trials implementing quadruple-blinding possess robust infrastructure and stringent oversight, thereby lowering their threshold for scrutinizing other design aspects, such as randomization specifics. Consequently, methodological sophistication in blinding may enhance the perceived credibility of a manuscript, facilitating its path to publication (23). These observations underscore that editorial decision-making is influenced not only by trial outcomes but also by disease context and methodological sophistication.

A notable paradox emerged from our multivariable analysis. Although industry-sponsored trials constituted the numerical majority of completed RCTs, non-industry funding was independently associated with a significantly higher likelihood of full-text publication. This inverse relationship between sponsorship prevalence and publication visibility likely reflects fundamental differences in dissemination behavior. Industry sponsors operate under stringent commercial confidentiality and competitive market pressures; negative or equivocal trial results are frequently sequestered as internal reports or regulatory submissions rather than submitted for peer review (24, 25). Conversely, non-industry trials—including academic and hybrid models—are driven primarily by career progression incentives and institutional mandates that prioritize public dissemination, regardless of effect size or commercial value (26). Consequently, the body of literature accessible to clinicians and researchers is inherently skewed: it overrepresents non-industry initiatives while underrepresenting the larger volume of industry-conducted trials. This selectivity challenges the assumption that published RCTs are representative of the totality of clinical evidence and may inadvertently amplify the perceived impact of smaller, non-industry studies while obscuring the full spectrum of industry-generated data.

Although most published literature met formal CONSORT requirements, the deconstruction of key methodological details revealed persistent substantive gaps in transparency. Low reporting rates for random sequence generation and allocation concealment directly impair the internal validity of RCTs. If a manuscript merely mentions “random allocation” without specifying the exact randomization algorithm (e.g., block randomization) or the allocation concealment mechanism (e.g., a central randomization system), the comparability between groups is theoretically unsound (27). Furthermore, the widespread lack of access to trial protocols may prevent readers from distinguishing predefined hypotheses from *post hoc* analyses. Such reporting deficiencies are particularly prominent in small-sample trials, reflecting insufficient training in methodological writing among some research teams and a failure to recognize that detailed procedural descriptions are prerequisites for the reproducibility of results (28).

Even in the published literature, significant design flaws exist. Approximately half of the trials exhibited at least one bias domain with high or unclear risk, with flaws most concentrated in “random sequence generation” and “allocation concealment” (29). This finding indicates that, in practice, some researchers may still use noncentralized, predictable grouping methods rather than truly random numbers generated by a computer. This phenomenon is likely due to these trials often being run independently by clinical departments that lack support from professional clinical pharmacology or biostatistics teams (30).

The incidence of the composite endpoint “research waste,” as defined in this study, was extremely high. Non-tumor diseases, domestic recruitment, a single-center design, and sample sizes less than 550 were associated with the highest risk of waste (31). This combination of characteristics suggests that trials lacking support from large-scale, multicenter collaborative networks and constrained by smaller sample sizes have a significantly higher failure rate throughout the process, from completion to successful publication. Conversely, the strong anti-waste properties demonstrated in international multicenter trials stem from the establishment of standard operating procedures (SOPs) and centralized randomization systems; this structured research model effectively mitigates the randomness that can arise from single-center operations. Additionally, studies initiated after 2010 showed a decreasing trend in waste, coinciding with the mandatory implementation of clinical trial registration regulations and the widespread adoption of the CONSORT statement, suggesting that external regulatory pressure and standardized operational models are key variables in promoting the closed-loop process of clinical trials from “registration” to “publication” (32–34).

To address the identified research waste, several strategies warrant consideration (35–37). Efforts to extend prospective trial registration and improve amendment justification—currently more rigorously applied to industry trials—could be beneficial for non-industry studies.​ Creating dedicated channels for publishing methodologically sound neutral or negative results, especially in non-tumor fields, may help counteract the current bias toward novelty.​ Enhancing methodological training for investigators conducting single-center trials might also improve adherence to standardized protocols.​ Finally, increasing the availability of lay summaries and clinical study reports could broaden access to trial findings beyond traditional publications. Taken together, these steps may foster a more complete and transparent evidence base.

This study has several limitations. First, the limited search terms may have resulted in the omission of some registered studies. Reliance on ClinicalTrials.gov may overlook trials on regional registries and overrepresent North American and industry-sponsored studies. Second, restrictions to phase III/IV trials likely lead to an underestimation of total research waste, as early-phase trials face higher discontinuation and non-publication rates. Third, despite a lag period, time-truncation bias may persist; defining “unpublished” through major bibliographic databases risks misclassifying trials that are published only as abstracts or preprints.​ Moreover, we utilized the original Cochrane Risk of Bias tool, appropriate for the era of trial conduct, but we will consider RoB 2.0 in future updates (14).​ ​Finally, given the exploratory nature of this study, all the candidate variables were included in the multivariable logistic regression model using the Enter method; however, the final sample size after complete-case analysis was relatively modest, which may have limited the statistical power to detect significant associations.

## Conclusion

Our study provides a comprehensive quantification of the immunotherapy RCT landscape. Despite the expansion of immunomodulatory strategies beyond oncology, more than three-quarters of registered trials are compromised by research waste. We identified a distinct risk profile in which non-tumor diseases, single-center designs, domestic recruitment, and small sample sizes are significantly associated with higher rates of non-publication, inadequate reporting, and methodological flaws. In contrast, multi-center and international collaborations demonstrate strong resilience against such inefficiencies. These findings underscore the urgent need to prioritize large-scale collaborative frameworks, particularly for non-tumor indications, and to enforce stricter adherence to CONSORT guidelines to ensure that the substantial resources invested in immunotherapy trials yield reliable, translatable evidence for clinical practice.

## Data Availability

The original contributions presented in the study are included in the article/**Supplementary Material**. Further inquiries can be directed to the corresponding authors.
