# Supplementary material for: Characteristics and research waste of immunotherapy randomized clinical trials: a cross-sectional analysis
Source: Front Immunol. 2026 Aug 17;17:1845591. doi: 10.3389/fimmu.2026.1845591 (PMC13526974; doi:10.3389/fimmu.2026.1845591)
Supplement: Supplementary file 1 [file DataSheet1.pdf]

Table S1 Characteristics of RCTs related to immunotherapy regarding the status of the CONSORT reports

Characteristic	Adequate reporting (n=62)	No. (%)	Inadequate reporting (n=12)	No. (%)	P
**Start date**					0.738
2000-2004	8	12.9%	1	8.3%	
2005-2009	19	30.6%	3	25.0%	
2010-2014	18	29.0%	4	33.3%	
2015-2019	11	17.7%	4	33.3%	
After 2019	6	9.7%	0	0.0%	
**Conditions**					0.339
Tumor	27	43.6%	3	25.0%	
Non-tumor	35	56.5%	9	75.0%	
**Primary purpose**					0.117
Treatment	58	93.6%	10	83.3%	
Prevention	4	6.5%	1	8.3%	
Other	0	0.0%	1	8.3%	
**Study design**					>0.999
Parallel group	59	95.2%	12	100.0%	
Non-Parallel group	3	4.8%	0	0.0%	
**No. of arms**					0.722
2	48	77.4%	11	91.7%	
3	8	12.9%	1	8.3%	
≥4	6	9.7%	0	0.0%	
**Blinding**					0.402
None/open label	17	27.4%	1	8.3%	
Single	3	4.8%	0	0.0%	
Double	12	19.4%	5	41.7%	
Triple	7	11.3%	1	8.3%	
Quadruple	23	37.1%	5	41.7%	
**Recruitment**					0.057
National	26	41.9%	9	75.0%	
International	36	58.1%	3	25.0%	
**No. of centers**					0.186
Single center	18	29.0%	6	50.0%	
Multicenter	44	71.0%	6	50.0%	
**No. of participants**					0.046
<550	36	58.1%	11	91.7%	
≥550	26	41.9%	1	8.3%	
**Region of PI**					0.758
North America	18	29.0%	2	16.7%	
Europe	37	59.7%	9	75.0%	
Asia	4	6.5%	1	8.3%	
Others	3	4.8%	0	0.0%	
**Funding**					0.107
Industry	39	62.9%	4	33.3%	
Non-industry	23	37.1%	8	66.7%	

PI: principal investigator.

Table S2 Compliance with items in CONSORT 2010 Checklist

	CONSORT Item	Percent
**1a**	Identification as a randomised trial in the title	98.6% (73/74)
**1b**	Structured summary of trial design, methods, results, and conclusions	98.6% (73/74)
**2a**	Scientific background and explanation of rationale	98.6% (73/74)
**2b**	Specific objectives or hypotheses	91.9% (68/74)
**3a**	Description of trial design (such as parallel, factorial) including allocation ratio	97.3% (72/74)
**3b***	Important changes to methods after trial commencement (such as eligibility criteria), with reasons	-
**4a**	Eligibility criteria for participants	90.5% (67/74)
**4b**	Settings and locations where the data were collected	100.0% (74/74)
**5**	The interventions for each group with sufficient details to allow replication	98.6% (73/74)
**6a**	Completely defined pre-specified primary and secondary outcome measures	94.6% (70/74)
**6b***	Any changes to trial outcomes after the trial commenced, with reasons	-
**7a**	How sample size was determined	78.4% (58/74)
**7b***	When applicable, explanation of any interim analyses and stopping guidelines	-
**8a**	Method used to generate the random allocation sequence	64.9% (48/74)
**8b**	Type of randomisation; details of any restriction (such as blocking and block size)	79.7% (59/74)
**9**	Mechanism used to implement the random allocation sequence	63.5% (47/74)
**10**	Who generated the random allocation sequence, enrolled participants, and assigned participants to interventions	70.3% (52/74)
**11a**	If done, who was blinded after assignment to interventions and how	75.7% (56/74)
**11b***	If relevant, description of the similarity of interventions	-
**12a**	Statistical methods used to compare groups for primary and secondary outcomes	93.2% (69/74)
**12b***	Methods for additional analyses, such as subgroup analyses and adjusted analyses	-
**13a**	The numbers of participants who were randomised, received treatment, and analysed for the primary outcome	94.6% (70/74)
**13b**	For each group, losses and exclusions after randomisation, together with reasons	91.9% (68/74)
**14a**	Dates defining the periods of recruitment and follow-up	96.0% (71/74)
**14b***	Why the trial ended or was stopped	-
**15**	A table showing baseline demographic and clinical characteristics for each group	91.9% (68/74)
**16**	For each group, number of participants analysed and whether the analysis was by original assigned groups	100.0% (74/74)
**17a**	For each primary and secondary outcome, results for each group, and the estimated effect size and its precision	94.6% (70/74)
**17b**	For binary outcomes, presentation of both absolute and relative effect sizes is recommended	93.2% (69/74)

**18***	Results of any other analyses performed, including subgroup analyses and adjusted analyses	-
**19**	All important harms or unintended effects in each group	96.0% (71/74)
**20**	Trial limitations, addressing sources of potential bias, imprecision, and, if relevant, multiplicity of analyses	98.6% (73/74)
**21**	Generalisability (external validity, applicability) of the trial findings	93.2% (69/74)
**22**	Interpretation consistent with results, balancing benefits and harms, and considering other relevant evidence	94.6% (70/74)
**23**	Registration number and name of trial registry	97.3% (72/74)
**24**	Where the full trial protocol can be accessed, if available	31.1% (23/74)
**25**	Sources of funding and other support (such as supply of drugs), role of funders	100.0% (74/74)

*A conditional item for which not all manuscripts were scored.

CONSORT: Consolidated Standards of Reporting Trials

Table S3 Characteristics of RCTs related to immunotherapy regarding the situation of design flaws

Characteristic	Absence of design flaw (n=37)	No. (%)	Presence of design flaw (n=37)	No. (%)	P
**Start date**					0.084
2000-2004	1	2.7%	8	21.6%	
2005-2009	10	27.0%	12	32.4%	
2010-2014	12	32.4%	10	27.0%	
2015-2019	10	27.0%	5	13.5%	
After 2019	4	10.8%	2	5.4%	
**Conditions**					0.032
Tumor	20	54.1%	10	27.0%	
Non-tumor	17	45.9%	27	73.0%	
**Primary purpose**					0.674
Treatment	35	94.6%	33	89.2%	
Prevention	2	5.4%	3	8.1%	
Other	0	0.0%	1	2.7%	
**Study design**					>0.999
Parallel group	35	94.6%	36	97.3%	
Non-Parallel group	2	5.4%	1	2.7%	
**No. of arms**					0.701
2	31	83.8%	28	75.7%	
3	4	10.8%	5	13.5%	
≥4	2	5.4%	4	10.8%	
**Blinding**					0.245
None/open label	10	27.0%	8	21.6%	
Single	2	5.4%	1	2.7%	
Double	5	13.5%	12	32.4%	
Triple	6	16.2%	2	5.4%	
Quadruple	14	37.8%	14	37.8%	
**Recruitment**					0.005
National	11	29.7%	24	64.9%	
International	26	70.3%	13	35.1%	
Missing	0	0.0%	0	0.0%	
**No. of centers**					0.001
Single center	5	13.5%	19	51.4%	
Multicenter	32	86.5%	18	48.6%	
**No. of participants**					0.147
<550	20	54.1%	27	73.0%	
≥550	17	45.9%	10	27.0%	
**Region of PI**					0.016
North America	14	37.8%	6	16.2%	
Europe	17	46.0%	29	78.4%	
Asia	6	16.2%	2	5.4%	
**Funding**					0.059
Industry	26	70.3%	17	46.0%	
Non-industry	11	29.7%	20	54.1%	

PI: principal investigator.

Table S4 Domain-specific risk of bias judgments based on the Cochrane Risk of Bias tool

**Risk of Bias Domain**	**Low risk, n (%)**	**High risk, n (%)**	**Unclear risk, n (%)**
Sequence generation	56 (75.7)	10 (13.5)	8 (10.8)
Allocation concealment	48 (64.9)	17 (23.0)	9 (12.2)
Blinding of participants	64 (86.5)	10 (13.5)	0 (0.0)
Blinding of outcome assessment	53 (71.6)	13 (17.6)	8 (10.8)
Incomplete outcome data	63 (85.1)	11 (14.9)	0 (0.0)
Selective reporting	70 (94.6)	4 (5.4)	0 (0.0)
Other bias	49 (66.2)	17 (23.0)	8 (10.8)
Overall	37 (50.0)	30(40.5)	7 (9.5)

Table S5 Distribution of research waste subtypes

**Waste subtype**	**Assessment group**	**n (%) ***
Any research waste	All eligible RCTs (n=164)	127 (77.4)
Non-publication	All eligible RCTs (n=164)	90 (54.9)
Inadequate reporting	Published RCTs (n=74)	12 (16.2)
Avoidable design flaw	Published RCTs (n=74)	37 (50.0)
Both reporting and design flaw	Published RCTs (n=74)	12 (16.2)

*Research waste was defined as the presence of at least one of: non-publication, inadequate reporting, or avoidable design flaw. Inadequate reporting and avoidable design flaw were assessed only among published RCTs; therefore, percentages for these subtypes are calculated relative to the published subgroup.
